# Study on the relationship between social support, health information-seeking behavior, and health literacy among rural patients with diabetes: a cross-sectional study

**DOI:** 10.3389/fendo.2026.1921954

**Published:** 2026-10-22

**Authors:** Jingfeng Chen, Xiaoxue Lei, Chaoqun Bai, Jiaxia Han, Guifen Fu, Yanping Zhang

**Affiliations:** 1 Department of Geriatric Endocrinology and Metabolism, Guangxi Academy of Medical Sciences and the People’s Hospital of Guangxi Zhuang Autonomous Region, Nanning, Guangxi, China; 2 Department of Endocrinology and Metabolism, Guangxi Academy of Medical Sciences and the People’s Hospital of Guangxi Zhuang Autonomous Region, Nanning, Guangxi, China; 3 Department of Nursing, Guangxi Academy of Medical Sciences and the People’s Hospital of Guangxi Zhuang Autonomous Region, Nanning, Guangxi, China

**Keywords:** Diabetes mellitus, health information seeking behavior, health literacy, rural population, social support

## Abstract

**Objective:**

To explore the current status and relationships among health literacy, social support, and health information-seeking behaviors among rural patients with diabetes and to provide a basis for developing targeted interventions to improve health management in this population.

**Setting:**

Fifteen county-level hospitals across five cities (Nanning, Guilin, Hechi, Chongzuo, and Yulin) in the Guangxi Zhuang Autonomous Region of China. The study was reported in accordance with the STROBE (Strengthening the Reporting of Observational Studies in Epidemiology) guidelines.

**Participants:**

Rural patients with diabetes (2,280) were recruited using a multistage stratified random sampling method between January and July, 2022. A total of 2,178 valid questionnaires were included in the final analysis.

**Primary and secondary outcome measures:**

The primary outcome measures were total scores on the Social Support Rating Scale (SSR), Diabetes Health Information-Seeking Behavior Scale (HISB), and Health Literacy Scale (HLS). The secondary outcome was the mediating effect of health information-seeking behaviors, analyzed using structural equation modeling (SEM).

**Results:**

The total scores for health literacy, social support, and health information-seeking behavior were (30.47 ± 9.36), 22 (IQR: 22, 30), and (109.45 ± 18.50), respectively. Spearman’s correlation analysis revealed significant positive correlations between all three variables (all *p* < 0.01). SEM analysis showed that social support was directly associated with health literacy (effect = 0.285, 95% CI: 0.247 to 0.322) and had a significant indirect association through health information-seeking behavior (effect = 0.160, 95% CI: 0.142 to 0.179). The mediating effect accounted for 36.0% of the total effect. The model fit indices were satisfactory (GFI = 0.958, AGFI = 0.935, CFI = 0.950, RMSEA = 0.072).

**Conclusions:**

Social support, health information-seeking behavior, and health literacy were positively correlated among rural patients with diabetes, with health information-seeking behavior partially mediating the relationship between social support and health literacy. These findings suggest that interventions targeting the enhancement of social support and the promotion of active health information-seeking may be promising approaches to improving health literacy in this population.

## 1 Introduction

The prevalence of type 2 diabetes mellitus (T2DM) has become a global health concern. According to projections by the International Diabetes Federation (IDF), approximately 783 million people worldwide will have diabetes by 2045. While T2DM prevalence is rising rapidly in developed countries, the increase is also notable in developing nations such as China (1, 2). By 2045, the number of people with diabetes in China is expected to reach 174 million, with rural areas experiencing a greater rate of increase than urban areas (3). A survey found that the prevalence of diabetes among rural adults in Guangxi was 5.9% (4). This high prevalence may be attributed to factors such as an aging population, obesity, and sedentary lifestyle (5, 6). Rural populations face unique challenges in diabetes management, including limited access to healthcare resources, lower socioeconomic status, restricted digital accessibility, and fewer channels for obtaining health information compared to urban populations (4, 10). These barriers may exacerbate health disparities and complicate diabetes self-management in rural settings, highlighting the need for targeted investigations in this population.

T2DM requires long-term management and is often accompanied by complications and comorbidities. Diabetes is a major public health challenge worldwide owing to its high rates of disability and mortality (7). Effective blood glucose control is crucial for the prevention of diabetic complications (8). Health literacy, defined as the ability to obtain, process, and understand basic health information and services needed to make appropriate health decisions, plays a pivotal role in the management of diabetes (9). However, studies have shown that the diabetes awareness rate among rural adults in Guangxi is only 11.8%, and the overall health literacy levels are low (4). Poor health literacy can result in insufficient health-related knowledge and self-management of the disease, further increasing the risk of complications (10). Health information seeking behavior is fundamental to making informed health decisions and is a key factor in improving health literacy. Patients with higher levels of social support are more likely to access, utilize, and comprehend health information, leading to better health management capabilities and improved health literacy (11). This proposed pathway is consistent with the Health Belief Model, which suggests that health-related behaviors are shaped by individuals’ perceptions and external cues to action. In this context, social support may act as a cue that motivates patients to actively seek health information, thereby enhancing their understanding and use of health information (12). Health information-seeking behavior may therefore serve as a mediating pathway through which social support influences health literacy. Understanding this mechanism is important for developing effective interventions to promote health literacy in rural populations. Despite the established theoretical links, most existing studies have been conducted in urban populations, with limited attention to rural settings where health literacy is lower and information access is more constrained (4, 10). Few studies have specifically examined the mediating role of health information-seeking behavior between social support and health literacy among rural patients with diabetes in Guangxi. This study was therefore conducted to address this gap.

This study aimed to investigate the relationships between social support, health information-seeking behavior, and health literacy among rural patients with diabetes. Based on the previous literature and theoretical models, we proposed the following hypotheses:

1. Social support is positively associated with both health literacy and health information-seeking behavior.
2. Health information-seeking behaviors are positively associated with health literacy.
3. Health information-seeking behavior mediates the relationship between social support and health literacy.

## 2 Materials and methods

### 2.1 Study design

This study employed a cross-sectional design to examine the relationships between social support, health information-seeking behavior, and health literacy in rural patients with diabetes. This study was conducted between January and July 2022 in the Guangxi Zhuang Autonomous Region, China.

### 2.2 Participants

This study used a multistage stratified sampling method to select participants between January and July, 2022. In the first stage, five cities in the Guangxi Zhuang Autonomous Region were randomly selected based on their geographic regions (central, eastern, southern, western, and northern): Nanning, Guilin, Hechi, Chongzuo, and Yulin. In the second stage, three counties were randomly selected from each city, resulting in a total of 15 counties. One hospital with an internal medicine department was chosen from each county for the survey. The sample size was calculated using the formula for cross-sectional studies: n = Z²_1_-α/_2_ × P(1−P)/δ², where Z_1_-α/_2_ = 1.96 (for a 95% confidence level), P = 0.773 (the estimated prevalence of diabetes-related complications based on a pilot survey of 150 patients in the same setting), and δ = 0.1 (the acceptable margin of error). A design effect of 2.0 was applied to account for the multistage sampling design, yielding a minimum of 136 participants per county. After adding 10% to account for potential non-response or incomplete questionnaires, the final target was 152 participants per county. A total of 2,280 patients were included in the study. The inclusion criteria were as follows: (1) meeting the 2020 diagnostic criteria for diabetes set by the Chinese Diabetes Society (13).(2)Age ≥ 18 years. (3) Registered household and current residence in the selected study areas. The exclusion criteria were as follows: (1) patients with gestational diabetes. (2) History of dementia or other psychiatric disorders as determined by medical records or family interviews. (3) Patients in the acute phase of their illness who are unable to participate in the survey. The participants were selected using a multistage stratified random sampling method from county-level hospitals across five regions in Guangxi to ensure geographic and socioeconomic diversity within the sample of rural patients with diabetes. All hospital administrators involved in the study provided informed consent and all participants signed written informed consent forms. The study protocol was approved by the Ethics Committee of the Guangxi Zhuang Autonomous Region People’s Hospital (Approval Number: KT-KJT-2021-26).

### 2.3 Survey instruments

#### 2.3.1 General information questionnaire

A general information questionnaire was developed by the researchers. It included demographic data such as age, sex, marital status, educational level, and per capita annual household disposable income (14). Disease-related information included illness duration, glycated hemoglobin (HbA1c) levels, use of antidiabetic medications, and use of insulin therapy.

#### 2.3.2 Diabetes health information seeking behavior

The Health Information Seeking Behavior (HISB) scale, originally developed by Sofia (15) was translated and revised into Chinese by Wang Meifeng et al. (16). It reflects the health information needs of diabetes patients and consists of eight dimensions: information needs, access through healthcare professionals, access through books, access through the Internet, interpersonal communication, subjective barriers to information, objective barriers to information, and cultural barriers. The scale includes 38 items with a total score ranging from 38 to 190. A 5-point Likert scale was used, where higher scores indicated more prominent health information-seeking behavior. The Chinese version of the HISB scale demonstrated good validity and reliability, with a content validity index of 0.92 and a Cronbach’s alpha of 0.866. The full-scale items are provided in **Supplementary File 1**.

#### 2.3.3 Health literacy scale

The Health Literacy Scale (HLS) is an assessment tool developed by Ishikawa et al. in 2008 (17) to evaluate health literacy among patients with diabetes. In 2021, Xiaoyan et al. (18) translated and validated the scale for use in the Chinese population. The HLS consists of three dimensions: functional health literacy, communicative health literacy, and critical health literacy, with a total of 14 items that use a 4-point Likert scoring system ranging from 1 (“not at all”) to 4 (“most of the time”). Functional health literacy dimensions were reverse-scored. The total score ranged from 14 to 56, with higher scores indicating better health literacy levels. The Cronbach’s alpha coefficient of the Chinese version of the HLS is 0.868, indicating good reliability. The full-scale items are provided in **Supplementary File 2**.

#### 2.3.4 Social support

The Social Support Rate Scale (SSR), developed by Xiao Shuiyuan (19), is used to evaluate social support levels. It includes three dimensions: subjective, objective, and support utilization. The scale consists of ten items. Items 1–5 and 8–10 are single-choice questions scored using a 4-point scale. For items 6 and 7, the response of ‘no source’ was scored as 0, while the number of sources reported corresponded to the score, with a total scores ranging from 12 to 66, and higher scores indicating better levels of social support. The SSR has demonstrated excellent reliability, with a Cronbach’s alpha coefficient ranging from 0.89 to 0.94 and a test-retest reliability of 0.92. The full-scale items are provided in **Supplementary File 3**.

### 2.4 Survey methods

Before the formal survey, investigators from 15 hospitals underwent standardized training. They explained the study’s purpose and content, estimated the completion time to the participants, and strictly adhered to confidentiality principles. The questionnaire contained a large number of items. To minimize respondent fatigue and ensure data quality, trained investigators provided onsite guidance during completion and checked each questionnaire immediately after submission to ensure completeness and accuracy. The average completion time was maintained at 30 min. Disease-related data were obtained from the patients’ electronic medical records.

### 2.5 Statistical methods

In this study, social support was defined as the independent variable, health literacy as the dependent variable, and health information-seeking behavior as the mediating variable. The hypothesized structural model aimed to assess both the direct and indirect effects of social support on health literacy through health information-seeking behavior.

Data were double-entered and cross-verified using Microsoft Excel 2020. Incomplete questionnaires were excluded from the analysis. Normality of continuous variables was assessed using the Kolmogorov–Smirnov test. Normally distributed variables are presented as mean ± standard deviation (SD), and non-normally distributed variables are presented as median with interquartile range (IQR). Statistical analyses were performed using SPSS 22.0. A structural equation model (SEM) was constructed using AMOS 24.0. Before model fitting, sample size adequacy was confirmed, multivariate normality was assessed (with robust estimation applied due to minor deviations), and no evidence of multicollinearity was found (all VIFs < 3.0). These checks ensured that the assumptions for SEM were adequately met. The model was refined based on modification indices (MI) and substantive reasoning. Residual covariances were added only where MI values exceeded the conventional threshold (MI > 3.84) and where items shared conceptual overlap or belonged to the same theoretical subscale; these additions were kept to a minimum to avoid overfitting. The “information needs” dimension was removed due to consistently low factor loadings and non-significance. The bootstrap method with 5,000 resamples was employed to test for mediation effects. A significance level of α = 0.05 was used for all statistical tests.

## 3 Results

### 3.1 General information of participants

A total of 2,280 rural patients with diabetes were surveyed, of whom 2,178 (95.5%) provided valid responses and were included in the final analysis. The remaining 102 questionnaires (4.5%) were excluded due to incomplete responses or missing data. The average age of the participants was (63.25 ± 12.71) years, and the average disease duration was(7.96 ± 4.07)years. Additional demographic and clinical characteristics of the patients are shown in **Table 1**.

**Table 1:** General characteristics of rural patients with diabetes surveyed (n = 2178).

Characteristic	Classification	Value(Percentage, %)
Sex	Male	1204(55.3)
	Female	974(44.7)
Marital Status	Married	2020(92.7)
	Unmarried or Widowed	158(7.3)
Education Level	Less than Primary School	256(11.7)
	Primary School	504(23.1)
	Junior High School	684(31.4)
	High School	507(23.3)
	Specialized Degrees	180(8.3)
	Bachelor Degree or Above	47(2.2)
Disposable Annual Household Income per Capita	<18931 RMB	1356(62.3)
	≥18931 RMB	822(37.7)
Family History of Diabetes	Yes	317(14.6)
	No	1861(85.4)
HbA1c Control Status	<7.0	494(22.7)
	≥ 7.0	1684(77.3)

### 3.2 Scores of social support, health information seeking behavior, and health literacy among rural patients with diabetes

The total health literacy score of rural diabetes patients was 30.47 ± 9.36. The scores of its three dimensions, ranked from highest to lowest, were functional health literacy (11.17 ± 3.76), communicative health literacy (10.64 ± 3.92), and critical health literacy (8.65 ± 3.07). The total social support score was 22 (IQR: 22, 30), with scores for the following three dimensions: subjective support 8 (IQR:7, 13), objective support 7 (IQR: 5, 12), and support utilization 7 (IQR:6, 8). The total score for health information-seeking behaviors was 109.45 ± 18.50. The scores of its eight dimensions, in descending order, were information needs (34.35 ± 6.44), healthcare professional channels (18.92 ± 4.43), interpersonal communication channels (12.63 ± 3.10), cultural level (11.27 ± 2.84), book channels (10.46 ± 3.27), objective barriers (8.75 ± 2.24), subjective barriers (8.62 ± 1.96), and internet channels (4.44 ± 1.61).

### 3.3 Correlation analysis among social support, health information-seeking behavior, and health literacy in rural patients with diabetes

The total health literacy score was positively correlated with the total scores for social support and health information-seeking behavior (r = 0.482, r = 0.513, both *p* < 0.001). Additionally, the total social support score was positively correlated with the total score for health information-seeking behavior (r = 0.503, *p* < 0.001).

### 3.4 Model evaluation and path analysis of social support, health information seeking behavior, and health literacy in rural patients with diabetes

Based on the research hypothesis, a structural equation model was constructed with social support as the exogenous latent variable, health information-seeking behavior as the mediating latent variable, and health literacy as the outcome variable. The “information needs” dimension was removed due to low factor loadings and non-significance, and residual covariances were added where supported by both modification indices and content overlap (see Statistical Methods for details). The revised model demonstrated acceptable fit: GFI = 0.958, AGFI = 0.935, CFI = 0.950, and RMSEA = 0.072. Mediation effect analysis using the bootstrap method revealed that the path coefficients were statistically significant, supporting the research hypothesis. All reported path coefficients are standardized. As shown in **Table 2**, social support had a significant direct effect on health information-seeking behavior (β = 0.859, 95% CI: 0.798 to 0.920, p < 0.001), and health information-seeking behavior in turn had a significant direct effect on health literacy (β = 0.186, 95% CI: 0.168 to 0.204, p < 0.001). The direct effect of social support on health literacy was 0.285 (95% CI: 0.247 to 0.322), and the indirect effect via health information-seeking behavior was 0.160 (95% CI: 0.142 to 0.179). The indirect effect accounted for 36.0% of the total effect, indicating partial mediation. The structural equation model is shown in **Figure 1**.

**Table 2:** Results of the mediating effect of social support and health information seeking behavior on health literacy among rural patients with diabetes.

Paths	*P*	Direct effect	95%CI	Indirect effect	95%CI
Social support→Health literacy	< 0.001	0.285	0.247˜0.322	0.160	0.142˜0.179
Social support→Health information seeking behavior	<0.001	0.859	0.798˜0.920	–	–
Health information seeking behavior→Health literacy	< 0.001	0.186	0.168˜0.204	–	–

**Figure 1:**
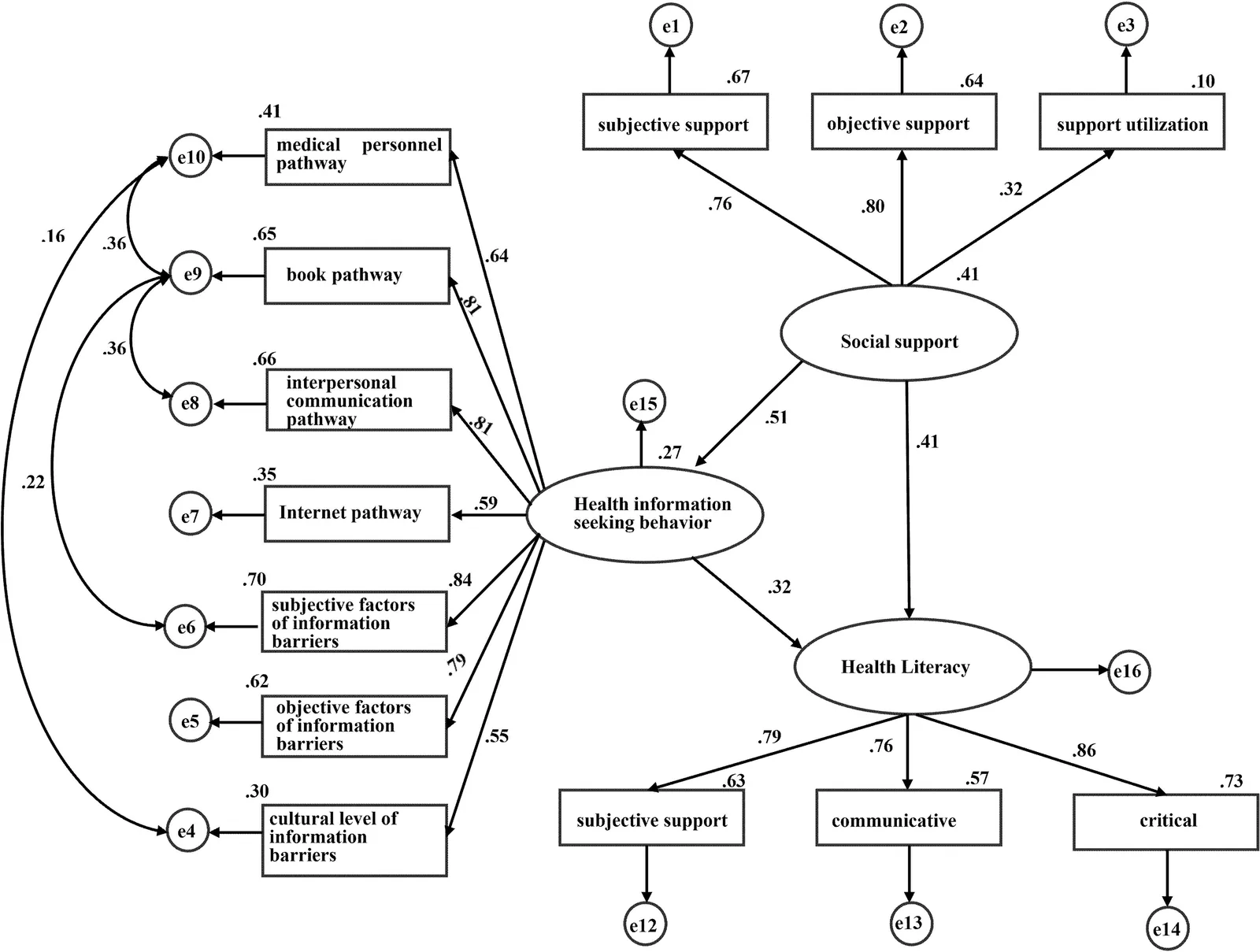
Structural equation model showing the associations among social support, health information-seeking behavior, and health literacy in rural patients with diabetes. Standardized path coefficients are displayed on the arrows. Solid lines indicate statistically significant paths (*p* < 0.05). SSR, social support; HISB, health information-seeking behavior; HL, health literacy.

## 4 Discussion

### 4.1 Current status of social support, health information-seeking behavior, and health literacy among rural patients with diabetes

Health literacy is a crucial nonclinical factor that reduces the risk of adverse outcomes in diabetes management (20). The results of this study indicated that the average health literacy score of rural diabetes patients was 35 (30.47 ± 9.36), suggesting that the health literacy of these patients is at a moderate level. This finding is consistent with research by AnYiqing et al. (21), but lower than the results of Liu Teng Teng et al. (22), who studied the health literacy levels of patients with diabetes in communities in Hangzhou. This difference may be due to the inclusion of a higher proportion of middle-aged and older patients in this study. As age increases, the ability to access and understand information decreases, which may contribute to lower health literacy levels. Previous studies have shown that patients with low health literacy often struggle with reading and comprehension skills, which can hinder their ability to manage their health, understand prescriptions, and make informed decisions about healthcare resources. These barriers can result in poor health outcomes (23). Among the health literacy dimensions, functional health literacy scored highest, whereas critical health literacy scored lowest. This suggests that rural patients with diabetes have insufficient knowledge about diabetes, which likely affects their ability to critically evaluate health information (24). Consequently, overall health literacy among rural patients with diabetes remains suboptimal.

The results showed that the health information-seeking behavior score of rural diabetes patients was 124 (109.45 ± 18.50), indicating that their health information-seeking behavior is at a moderate level. This finding is similar to that of a study by Nina et al. (25) on health literacy in community diabetic populations. Patients with higher health information-seeking behavior scores obtained more effective health information, which enhanced their ability to engage in diabetes self-management. In this study, the information needs dimension had the highest score, while the Internet channel had the lowest score, suggesting that rural patients with diabetes have insufficient health knowledge and limited sources of information. These findings indicate that rural patients with diabetes have limited sources of health information. While our study did not directly test the effectiveness of any intervention, these observations suggest that assisting patients in acquiring sufficient, reliable, and applicable health information—and potentially integrating new technologies such as telemedicine—may be worth exploring in future efforts to improve health literacy and self-management. It is worth noting that although the “information needs” dimension scored highest in the descriptive analysis, it was removed from the final SEM due to poor factor loadings and non-significance. This discrepancy may partly reflect the characteristics of our rural sample, in which information needs may be passive or unrecognized rather than actively pursued. Future studies using qualitative methods could help clarify how rural patients with diabetes perceive and articulate their health information needs.

The results showed that the social support score for rural diabetes patients was 22 (IQR:22–30), indicating a relatively low level i.e., lower than the social support level for rural type 2 patients with diabetes in Henan Province (26). Social support has long been considered as a necessary and important component of ongoing diabetes treatment (27). Social support from family, friends, colleagues, and other community members can promote patients’ self-management behaviors through both subjective and objective forms of support (28). This study also found that rural patients with diabetes scored lower on support utilization and objective factors. Healthcare providers should consciously integrate various resources such as professional lectures, volunteer clinics, and patient experience-sharing sessions to provide strong social support to patients.

### 4.2 The relationship between social support, health information seeking behavior, and health literacy in rural patients with diabetes

This study found that social support showed a direct positive association with health literacy and with health information-seeking behavior, and that health information-seeking behavior was positively associated with health literacy. These findings are consistent with our research hypotheses. The level of social support for patients with diabetes was positively associated with their health information-seeking behavior, which in turn was positively associated with their health literacy. This finding suggests that strengthening social support may contribute to improving patients’ ability to obtain health information and potentially enhance their health literacy, although residual confounding from unadjusted covariates cannot be ruled out given the cross-sectional design. Consistent with our finding that social support was positively associated with health literacy, these results suggest that interventions aimed at strengthening both subjective and objective support may be beneficial. However, our cross-sectional data cannot confirm that enhancing social support would directly improve health literacy; future interventional studies are needed to test this possibility. Diabetes management is a long and complex process, and the key factors influencing health literacy are the ability to communicate and access health information closely related to blood sugar control. Therefore, interventions that strengthen social support, expand patients’ health information channels, and encourage the acquisition of effective health information may be beneficial for improving health literacy, supporting blood glucose control, and potentially reducing the risk of complications.

### 4.3 Health information seeking behavior as a partial mediator between social support and health literacy in rural patients with diabetes

The mediation analysis revealed that health information-seeking behavior partially mediated the relationship between social support and health literacy in rural patients with diabetes. This finding suggests that social support was positively associated with health information-seeking behavior, which in turn was positively associated with health literacy in this population.Patients with diabetes need to follow a continuous self-management plan, which includes self-regulating their diet, controlling blood glucose levels, engaging in physical exercise, managing medications (including prescribed dosages, administration frequency, and timing), and practicing foot care (29). These self-management practices play a critical role in effective treatment of diabetes (30). However, patients with lower health literacy may experience difficulties in understanding health-related information and struggle to communicate their conditions to healthcare providers, leading to poorer self-management abilities (31).Therefore, interventions aimed at enhancing social support and promoting health information-seeking behavior may be effective approaches for improving health literacy among rural patients with diabetes. With technological advancements, information management platforms are gradually being applied in diabetes management. These platforms can provide patients with effective health information resources, thereby improving health literacy (32). Although our study was not designed to evaluate digital interventions, the finding that the internet channel scored lowest among all HISB dimensions suggests that improving access to reliable online health information may be a promising avenue for future interventions. However, this recommendation should be regarded as hypothesis-generating rather than definitive, and its effectiveness requires testing in longitudinal or interventional studies. This study highlights the positive associations of social support and health information-seeking behavior with health literacy among rural patients with diabetes. Future research should adopt longitudinal and interventional designs to explore the causal relationships between these variables and to test the effectiveness of targeted nursing or community-based interventions. Expanding the research scope to include different regions and populations would also help validate the generalizability of the findings. It should be noted that the cross-sectional design does not establish temporal ordering or causal mediation. The observed indirect pathway should therefore be interpreted as a statistical association consistent with the hypothesized model, rather than definitive evidence of causation. While our findings suggest potential avenues for intervention, integrating digital health technologies and telemedicine platforms into diabetes management warrants further investigation to determine their effectiveness in enhancing health literacy and self-management in rural settings.

These findings are broadly consistent with previous research. A study from the same rural population in Guangxi also reported significant associations between diabetes knowledge and health information-seeking behavior (33). Similar mediation pathways have been reported in other chronic disease contexts; for example, a study among pregnant women with gestational diabetes found that e-health literacy mediated the relationship between social support and behavioral decision-making (34). The use of the Health Belief Model as a theoretical framework is also supported by prior work integrating this model with social support theory to explain health information seeking (35). However, the magnitude of the observed indirect effect may vary across populations, as one study found that e-health literacy and patient-physician communication moderated the impact of online information seeking on self-care behaviors (36). These comparisons should be interpreted with caution given the cross-sectional design of our study.

## 5 Limitations

This multicenter cross-sectional study was conducted through surveys at 15 county-level hospitals. Although a multistage stratified random sampling method was used, the sample was drawn exclusively from patients attending county-level hospitals in Guangxi, a single province in China. This may limit generalizability, as the cultural, socioeconomic, and healthcare contexts in Guangxi may differ from other rural regions. Additionally, patients who do not regularly access healthcare services—particularly those in remote villages with limited mobility or lower health awareness—were not represented, potentially introducing selection bias. Therefore, our findings should be extrapolated to broader rural populations with caution, and future studies incorporating diverse geographic regions and community-based recruitment are warranted. We used the Social Support Scale, Health Information-Seeking Behavior Scale, and HLS to assess patients’ conditions. However, these tools may require a certain amount of time to be administered in clinical settings. Although a multistage stratified random sampling method was used, the sample was drawn exclusively from patients who visited county-level hospitals in Guangxi, China. Therefore, the participants may not fully represent all rural residents with diabetes, particularly those with limited access to medical care or those who do not seek hospital services. This potential limitation should be considered when generalizing the findings to a larger rural population with diabetes. Additionally, potential confounders—including age, education, income, diabetes duration, HbA1c, and insulin use—were not adjusted for in the SEM to preserve model parsimony. Residual confounding cannot therefore be excluded, and findings should be interpreted with caution. Additionally, all key variables were self-reported and collected at a single time point, which may introduce common method bias. We did not assess this statistically, and the possibility of inflated associations cannot be ruled out. Finally, the cross-sectional design precludes establishing causality or temporal mediation. Reliance on self-reported measures may introduce social desirability bias. These limitations should be considered when interpreting the findings.

## 6 Conclusion

This study demonstrated that social support, health information-seeking behavior, and health literacy were positively correlated among rural patients with diabetes. Social support showed both a direct association with health literacy and an indirect association through health information-seeking behavior, which partially mediated this relationship. These findings suggest that strengthening social support and promoting health information-seeking behavior may be important strategies for improving health literacy among rural diabetic populations. Therefore, health care providers should actively integrate community and family resources, develop accessible health information channels, and strengthen diabetes education to improve patients’ overall disease management and quality of life.

## 7 Strengths and limitations of this study

1. This study used a multicenter design with a multistage stratified random sampling method across 15 county-level hospitals.
2. Data were collected using validated scales, including the Social Support Rating Scale and the Health Information Seeking Behavior Scale.
3. SEM was employed to test the complex mediating relationships outlined in the research hypothesis.
4. This cross-sectional design limits the ability to infer causal relationships between the variables.
5. The generalizability of the findings may be limited because the sample was drawn exclusively from hospital patients.

## Data Availability

The original contributions presented in the study are included in the article/**Supplementary Material**. Further inquiries can be directed to the corresponding author.
