# Supplementary material for: Study on the relationship between social support, health information-seeking behavior, and health literacy among rural patients with diabetes: a cross-sectional study
Source: Front Endocrinol (Lausanne). 2026 Oct 22;17:1921954. doi: 10.3389/fendo.2026.1921954 (PMC13735596; doi:10.3389/fendo.2026.1921954)
Supplement: Supplementary file 1 [file SupplementaryFile1.docx]

**Health Information Seeking Behavior Scale**

Guideline: The following questions are used to undeGrstand your health information seeking behavior. Please choose the one that is closest to your actual situation among the five answers after each question and tick the corresponding option.

| How important do you think the following entries are in motivating you to seek information about diabetes? (Please select an appropriate answer) | Unimportant | Kind of important | Quite important | Very important | Extremely important |
| --- | --- | --- | --- | --- | --- |
| 1. What is diabetes? | 1 | 2 | 3 | 4 | 5 |
| 2.What are the symptoms of diabetes? | 1 | 2 | 3 | 4 | 5 |
| 3.What are the causes of diabetes? | 1 | 2 | 3 | 4 | 5 |
| 4.What are the complications of diabetes? | 1 | 2 | 3 | 4 | 5 |
| 5. What is a suitable diet for diabetics? | 1 | 2 | 3 | 4 | 5 |
| 6.What are the appropriate exercise methods for people with diabetes? | 1 | 2 | 3 | 4 | 5 |
| 7.What are the new drugs for diabetes treatment? What are the adverse effects of these drugs? | 1 | 2 | 3 | 4 | 5 |
| 8.When do I need to inject insulin? | 1 | 2 | 3 | 4 | 5 |
| 9.How should I use insulin? For example: type of insulin, injection site, method of injection, etc. | 1 | 2 | 3 | 4 | 5 |
| 10. What measures can be taken to prevent the development of diabetic foot? | 1 | 2 | 3 | 4 | 5 |
| 11.What are the symptoms of hypoglycemia? | 1 | 2 | 3 | 4 | 5 |
| 12. What should I do if there are other concomitant illnesses, such as infections? | 1 | 2 | 3 | 4 | 5 |
| What are your sources of information? (Please select an appropriate answer) | Have never | Infrequent | Now and then | Non-recurrent | Always |
| 13. Doctors | 1 | 2 | 3 | 4 | 5 |
| 14. Nurses | 1 | 2 | 3 | 4 | 5 |
| 15. Health followers | 1 | 2 | 3 | 4 | 5 |
| 16. Ophthalmologists | 1 | 2 | 3 | 4 | 5 |
| 17. Nutritionist | 1 | 2 | 3 | 4 | 5 |
| 18. Pharmacists | 1 | 2 | 3 | 4 | 5 |
| 19. Networking | 1 | 2 | 3 | 4 | 5 |
| 20. Books | 1 | 2 | 3 | 4 | 5 |
| 21. Leaflets | 1 | 2 | 3 | 4 | 5 |
| 22.Magazines, newspapers or news magazine journals | 1 | 2 | 3 | 4 | 5 |
| 23 Media (TV, radio) | 1 | 2 | 3 | 4 | 5 |
| 24.Including family or friends with diabetes | 1 | 2 | 3 | 4 | 5 |
| 25.Patient organizations or groups | 1 | 2 | 3 | 4 | 5 |
| 26. Other diabetics | 1 | 2 | 3 | 4 | 5 |
| 27.Diabetes-related lectures or seminars | 1 | 2 | 3 | 4 | 5 |
| 28.Books, pamphlets, etc. of clinics or health care personnel | 1 | 2 | 3 | 4 | 5 |
| How important do you think the following barriers are in preventing you from seeking knowledge about diabetes(Please select an appropriate answer) | Unimportant | kind of important | Quite important | Very important | Extremely important |
| 29. Lack of time | 1 | 2 | 3 | 4 | 5 |
| 30. Expenditure | 1 | 2 | 3 | 4 | 5 |
| 31. Lack of key infrastructure such as clinics, community health centers, etc. | 1 | 2 | 3 | 4 | 5 |
| 32. Lack of medical personnel | 1 | 2 | 3 | 4 | 5 |
| 33.Lack of computer literacy | 1 | 2 | 3 | 4 | 5 |
| 34.Large amount of cluttered and unorganized information | 1 | 2 | 3 | 4 | 5 |
| 35.Failure to understand relevant terminology | 1 | 2 | 3 | 4 | 5 |
| 36.Failure to understand information not in Chinese | 1 | 2 | 3 | 4 | 5 |
| 37.Doctor-patient relationship issues | 1 | 2 | 3 | 4 | 5 |
| 38.Psychosomatic problems | 1 | 2 | 3 | 4 | 5 |
