# Supplementary material for: Study on the relationship between social support, health information-seeking behavior, and health literacy among rural patients with diabetes: a cross-sectional study
Source: Front Endocrinol (Lausanne). 2026 Oct 22;17:1921954. doi: 10.3389/fendo.2026.1921954 (PMC13735596; doi:10.3389/fendo.2026.1921954)
Supplement: Supplementary file 2 [file SupplementaryFile2.docx]

**Health Literacy Scale**

Guideline: The following questions are used to understand your health literacy. Please choose the one that is closest to your actual situation among the four answers after each question and tick the corresponding option.

| In the past year, have you experienced any of the following when reading a hospital or pharmacy pamphlet or instruction manual? | Never | Seldom | Now and then | Most of the time |
| --- | --- | --- | --- | --- |
| 1. Found the font too small | 4 | 3 | 2 | 1 |
| 2. Words or phrases found to be unknown or unintelligible | 4 | 3 | 2 | 1 |
| 3. Content found too difficult to understand | 4 | 3 | 2 | 1 |
| 4. Takes a long time to read and understand | 4 | 3 | 2 | 1 |
| 5. Need someone to read for you | 4 | 3 | 2 | 1 |
| Since developing diabetes, have you had any of the following experiences of seeking information about diabetes (e.g., diagnosis, treatment, self-care, alternative treatments, etc.)? | Never | Seldom | Now and then | Most of the time |
| 6. Information can be gathered from a variety of sources | 1 | 2 | 3 | 4 |
| 7. You can quickly find the information you are looking for | 1 | 2 | 3 | 4 |
| 8. Ability to understand the information received | 1 | 2 | 3 | 4 |
| 9. Be able to share your ideas about health with others | 1 | 2 | 3 | 4 |
| 10. Ability to apply the information gained in everyday life | 1 | 2 | 3 | 4 |
| Did you have any of the following thoughts or behaviors when you obtained information about diabetes? | Never | Seldom | Now and then | Most of the time |
| 11. considered whether this information applies to your situation | 1 | 2 | 3 | 4 |
| 12. Consideration of the correctness of the information | 1 | 2 | 3 | 4 |
| 13. Verification of the correctness of this information | 1 | 2 | 3 | 4 |
| 14. Information gathering and health-related decision-making | 1 | 2 | 3 | 4 |
