# Supplementary material for: Study on the relationship between social support, health information-seeking behavior, and health literacy among rural patients with diabetes: a cross-sectional study
Source: Front Endocrinol (Lausanne). 2026 Oct 22;17:1921954. doi: 10.3389/fendo.2026.1921954 (PMC13735596; doi:10.3389/fendo.2026.1921954)
Supplement: Supplementary file 3 [file SupplementaryFile3.docx]

**Social Support Rate Scale**
GUIDELINE: The following questions are used to reflect the support you receive in the community, please fill in each question as specific to your situation. Thank you for your cooperation.
1. How many friends do you have who are close enough to get support and help? (check one only)
(1) None (2) 1 to 2
(3) 3 to 5 (4) 6 or more
2. In the past year, have you, (check one only)
(1) stayed away from your family and lived alone.
(2) Changed your place of residence frequently and lived with strangers most of the time.
(3) Lived with classmates, coworkers, or friends
(4) Lived with family
3. You and your neighbors: (check one only)
(1) Never care about each other, just nodding acquaintances.
(2) They may care a little when they are in trouble.
(3) Some neighbors care about you.
(4) Most of your neighbors care about you.
4. You and your coworkers: (check one only)
(1) Never care about each other, just nodding acquaintances.
(2) You may care a little when you are in trouble.
(3) Some coworkers care about you.
(4) Most coworkers care about you.
5. Support and care received from family members (tick the appropriate box)

|  | No support | Very little support | General Support | full support |
| --- | --- | --- | --- | --- |
| A.Couples (lovers) |  |  |  |  |
| B. parents |  |  |  |  |
| C.sons and daughters |  |  |  |  |
| D. siblings |  |  |  |  |
| E.Other members (e.g. sister-in-law) |  |  |  |  |

6. In the past, the sources of financial support or help in solving practical problems that you have received in times of emergency are:
(1) None of the sources.
(2) The following sources (check more than one)
A. Spouse B. Other family members C. Friends D. Relatives E. Colleagues F. Work unit G. Official or semi-official organizations such as political parties and trade unions H. Unofficial organizations such as religious and social groups I. Other (please list)
7. In the past, the sources of comfort and concern you used to receive when you were in an emergency situation were:
(1) No source
(2) The following sources: (check more than one)
A. Spouse B. Other family members C. Friends D. Relatives E. Colleagues F. Work unit G. Official or semi-official organizations such as party groups and trade unions H. Unofficial organizations such as religious and social groups I. Others (please list)
8. How do you talk to others when you are troubled: (Choose one only)
(1) Never tell anyone
(2) Only talk to 1 or 2 people who are very close to you.
(3) You will tell your friends if they ask.
(4) Taking the initiative to talk about your worries to get support and understanding
9. How do you seek help when you are in trouble: (check one only)

(1) Relying only on myself and not accepting help from others

(2) Rarely ask for help from others

(3) Sometimes ask others for help

(4) Often ask for help from family members, relatives, friends, and organizations when you are in trouble.

10. Regarding activities organized by groups (e.g., political parties and groups, religious organizations, trade unions, student unions, etc.), you (choose one only)

(1) Never participate

(2) Occasionally

(3) Often participate

(4) Take the initiative to participate and be active

Total points.

(i) Scoring method for Social Support Rating Scale entries

1. Questions 1 to 4, 8 to 10, one choice for each question, 1, 2, 3, 4 points for choosing 1, 2, 3, 4 items, respectively.

2. Question 5 is divided into five items, A, B, C, D, and E, and each item is scored from 1 to 4 points, from none to full support.

3. Questions 6 and 7 will be scored as 0 if the answer is "no source", but if the answer is "the following sources", the answer will be scored as many as there are sources.

(ii) Method of analyzing the social support scale

1. Total score: the sum of the scores of the 10 entries. 2.

2. objective support score: the sum of the scores for questions 2, 6 and 7. 3. subjective support score: the sum of the scores for questions 1, 2 and 3.

3. Subjective support score: the sum of the scores of questions 1, 3, 4 and 5. 4.

4. Utilization of support: the sum of the scores for questions 8, 9, and 10.
